# Metal Organic Framework@Polysilsesequioxane Core/Shell-Structured Nanoplatform for Drug Delivery

**DOI:** 10.3390/pharmaceutics12020098

**Published:** 2020-01-25

**Authors:** Liangyu Lu, Mengyu Ma, Chengtao Gao, Hongwei Li, Long Li, Fuping Dong, Yuzhu Xiong

**Affiliations:** 1 College of Materials and Metallurgy, Guizhou University, Guiyang 550025, China; gs.lylu17@gzu.edu.cn (L.L.); mmymamengyu@163.com (M.M.); chengtaogao@163.com (C.G.); lhwnoonoo123@163.com (H.L.); lilong@gzu.edu.cn (L.L.); 2 National Engineering Research Center for Compounding and Modification of Polymer Materials, Guiyang 550025, China

**Keywords:** metal organic framework, organosilica, polysilsesquioxane, drug delivery, surface functionalization

## Abstract

Modern pharmaceutics requires novel drug loading platforms with high drug loading capacity, controlled release, high stability, and good biocompacity. Metal–organic frameworks (MOFs) show promising applications in biomedicine owing to their extraordinarily high surface area, tunable pore size, and adjustable internal surface properties. However, MOFs have low stability due to weak coordinate bonding and limited biocompatibility, limiting their bioapplication. In this study, we fabricated MOFs/polysilsesquioxane (PSQ) nanocomposites and utilized them as drug carriers. Amine-functionalized MOF (UiO-66-NH_2_) nanoparticles were synthesized and encapsulated with epoxy-functionalized polysilsesquioxane layer on the surface via a facile process. MOFs possessed high surface area and regular micropores, and PSQs offered stability, inertness, and functionality. The obtained UiO-66-NH_2_@EPSQ nanocomposites were utilized as carriers for ibuprofen, a drug with carboxylic groups on the surface, and demonstrated high drug loading capacity and well-controlled release property. The UiO-66-NH_2_@EPSQ nanocomposite exhibited low cytotoxicity to HeLa cells within a wide concentration range of 10–100 µg/mL, as estimated by the MTT method. The UiO-66-NH_2_@EPSQ drug release system could be a potential platform in the field of controlled drug delivery.

## 1. Introduction

Development of a novel pharmaceutical platform with unique biological properties has gained tremendous academic interest in the field of nanobiomedicine [1,2]. Nanomaterials with a porous structure are widely investigated in biomedicine due to their features, such as suitable architecture, large surface area, and stability in biological fluids [3,4,5,6,7,8,9]. Among different types of porous materials, silica, calcium carbonate, calcium phosphate, and metal–organic frameworks (MOFs) have received significant attention in the last decade [10]. Although they are in the early stages of development [11,12], MOFs, wherein metal ions or clusters link organic ligands into porous materials, have shown great promise as a novel nanomedicine platform due to its large surface area, adjustable pore size, tunable host–guest interaction, and versatile functionality [13,14]. Preliminary biomedical applications of MOFs have focused on their use as delivery vehicles for molecular therapeutics and as viable contrast agents for optical imaging, multimodal imaging or X-ray computed tomography imaging [15,16]. In particular, MOFs are very promising nanoscale drug carriers due to their high molecule loading and easy functionalization [17,18,19,20,21,22]. MOFs have been utilized to deliver drug molecules through attachment of the prodrug onto the framework or direct use of the prodrug as a building block.

In pharmaceutical applications, enhancing the water stability, mechanical strength, and biocompatibility of MOFs while maintaining their other advantages remains a big challenge [23,24,25]. To solve this problem, researchers have attempted to fabricate MOF nanocomposites with other materials, such as polymers, silica, graphene, or carbon nanotubes, which have different properties, as the reinforcement phase [26,27,28,29,30,31,32,33,34,35,36,37,38]. Silica is suitable for preparation of composite materials to improve the performance of MOFs due to its high stability and structural adjustability [39,40,41,42]. Silica not only provides structural support for MOFs, but also improves the stability of materials through hydrophobic interaction or covalent bonding [43,44,45]. The modification of MOF surfaces with silica coatings improves the stability of MOFs, fine tunes their properties, and imparts additional functionality [46]. For example, Lin et al. first stabilized nano-MOFs by encapsulating them within a silica shell; the product offers several advantages, including biocompatibility, increased water dispersibility, and easy functionalization with silyl-derived molecules [47]. In detail, this study first coated MOF nanoparticles with PVP and treated them with tetraethylorthosilicate in basic ethanol to obtain MOF@silica core/shell nanostructures for controlled release of drugs. Sadr et al. also fabricated magnetic Fe_3_O_4_@silica@MIL-100(Fe) particles and modified the surface by grafting cyclodextrin for controlled delivery of cephalexin, an antibiotic drug [42]. Further modifications should be conducted to improve the stability, functionality, or biocompatibility of MOFs/SiO_2_ composites for drug delivery.

**Scheme 1:**
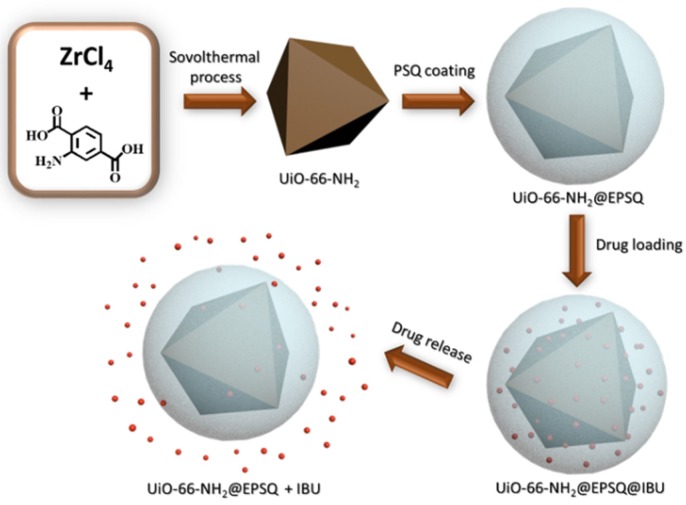
Preparation of UiO-66-NH_2_ and UiO-66-NH_2_@EPSQ for drug delivery.

In this work, a facile process was reported by encapsulating MOF nanoparticles with an organosilica layer. The product was directly utilized for drug delivery without further treatment. Polysilsesquioxanes (PSQs), an organosilica with organic/inorganic hybrid structure, have attracted much attention in the field of nanomedicine due to their high stability, biocompatibility, and easy functionalization [40,48]. We first fabricated UiO-66-NH_2_ nanoparticles, an amine-functionalized MOFs, from Zr ions and 2-aminoterephthalic acid ligand. We then coated epoxide-functionalized organosilica on UiO-66-NH_2_ particles with sodium lignosulfonate as stabilizer and ammonia as catalyst via a controlled sol–gel process in aqueous media (Scheme 1). PSQ layers, as a shell component, offer advantages such as good porous structure, high chemical stability, and good biocompatibility. UiO-66-NH_2_@EPSQ nanocomposites show high drug loading capacity and controlled release of ibuprofen, an anti-inflammatory drug. The MTT assay indicated that drug-free UiO-66-NH_2_@EPSQ nanocomposites demonstrated very low cytotoxicity. This study opens new opportunities to construct a safe and efficient delivery system for a wide range of applications.

## 2. Materials and Methods

### 2.1. Materials

2-Aminoterephthalic acid (98%) and polyvinylpyrrolidone (Mw avg. = 40,000) were purchased from Energy Chemical. (Energy Chemical, Shanghai, China). ZrCl_4_ (98%), sodium lignosulfonate, ammonia (AR, 25–28%), γ-(2,3-epoxypropoxy)propytrimethosysilane (97%), methanol (99.5%), ethanol (AR), Sodium chloride (for cell culture, for insect cell culture, ≥99.5%), Sodium phosphate dibasic dodecahydrate (GR, 99%), Potassium phosphate monobasic (for cell culture, for insect cell culture, ≥99%), Potassium chloride (for cell culture, ≥99.5%) and ibuprofen (GC, ≥98%) were supplied by Aladdin Reagents Co., Ltd. (Aladdin Reagents Co., Ltd., Shanghai, China). Acetic acid (AR, ≥99.5%) was purchased from Sinopharm Chemical Reagent Co., Ltd. (Sinopharm Chemical Reagent Co., Ltd, Shanghai, China). Fetal bovine serum was supplied by Bovogen Biological. (Bovogen Biologicals, C0230, Brazil). Dulbecco’s Modified Eagle’s Medium (DMEM) and trypsin-EDTA solution (0.25%, phenol red) were purchased from Invitrogen Gibco. (Invitrogen Gibco, Carlsbad, CA, US). Penicillin–streptomycin liquid (100×) and trypsin-EDTA were supplied by Beijing Solarbio Technology Co., Ltd. (Beijing Solarbio Technology Co., Ltd, Beijing, China). 3-(4,5-Dimethyl-2-thiazolyl)-2,5-diphenyl-2-H-tetrazolium bromide (MTT) was purchased from MedChemexpress CO., Ltd. (MedChemexpress CO., Ltd, HY-15924, NJ, US). All chemicals were used directly without further purification.

### 2.2. Characterization

Fourier transform infrared spectroscopy (FTIR) using KBr powder-pressed pellets with approximately 1 wt.% of the sample was recorded on a Perkin–Elmer Spectrum GX-spectrophotometer (Waltham, MA, US) with a spectral resolution of 1 cm^−1^ and scan number of 32. The morphology of all samples was observed by scanning electron microscopy (SEM) using a FEI-SEM system (FEI Helios Nanolab 600i, Hillsboro, OR, US) operating at 5 kV. For particle size estimation, more than 100 particles on the SEM images were averaged. Transmission electron microscopy (TEM) images were obtained using a FEI Tecnai G2F30 electron microscope (FEI, Hillsboro, OR, US) operating at 200 kV. The X-ray diffraction (XRD) pattern of the samples was recorded using a X PertPowder (PANalytical B.V., Almelo, Netherlands). The X-ray tube was operated at 40 kV and 40 mA (Cu Kα radiation with Ni filter, λ = 1.5406 Å). UV-visible spectra were recorded using the Evolution 201 (Thermo, Waltham, MA, US) UV-Visible spectrophotometer with 1 cm quartz cuvettes.

### 2.3. Synthesis of UiO-66-NH_2_@EPSQ Core/Shell Nanocomposite Materials

UiO-66-NH_2_ nanoparticles were prepared according to the procedure reported in literature [49]. Typically, 0.0628 g of ZrCl_4_ (0.34 mmol, 98%) and 0.0808 g of 2-aminoterephthalic acid (0.34 mmol, 98%) were added into blue-capped bottles with 100 mL of DMF. The bottle was then injected with 0.6 mL of acetic acid (glacial). The system was stirred for 24 h at 100 °C in an oil bath. After the reaction, the nanoparticles were separated by centrifugation and washed with DMF three times and with distilled water three times. Light brown UiO-66-NH_2_ nanoparticles were obtained after freeze drying.

The as-prepared UiO-66-NH_2_ nanoparticles (0.2 g) were dispersed in the mixture of 80 mL of methanol and 66 mL of water and ultrasonicated for 6 h. The system was combined with 0.2 g of sodium ligninsulfonate and 2 g of PVP and stirred for 10 min at room temperature. The suspension was transferred to an ice bath and stirred for 10 min. The suspension was combined with 4 mL of ammonia solution and dropwise with 1 mmol (for nanocomposites A), 2 mmol (for nanocomposites B), or 3 mmol (for nanocomposites C) γ-glycidyloxypropyltrimethoxysilane. The suspension was first stirred for 6 h in an ice bath and stirred for 12 h at room temperature. After the reaction, the nanoparticles were separated by centrifugation and washed three times with methanol and water. The final core/shell structured UiO-66-NH_2_@EPSQ nanocomposites were obtained after freeze drying.

### 2.4. Drug Loading and Release

Ibuprofen loading: According to the weight ratio of *W*_drug_/*W*_carrier_ listed in Table 1, a certain amount of ibuprofen and a corresponding amount of UiO-66-NH_2_@EPSQ nanoparticles were added into a vial containing 3 mL of ethanol and 7 mL of water. The system was first stirred for 6 h at 37 °C and stirred overnight at room temperature. The system was transferred to a dialysis bag with a molecular weight cut off of 3500 Da and immersed in 200 mL of deionized water for 12 h with gentle stirring at room temperature to form drug-loaded particle suspensions for the next step.

In vitro release: the dialysis bag was rinsed with water and then put into the PBS solution (pH 7.4). In intro drug release tests with ibuprofen on the UiO-66-NH_2_@EPSQ nanoparticles were performed with gentle stirring at room temperature. 3 mL of PBS solution was taken out from the released external solution at regular intervals, and then 3 mL of fresh PBS solution was added to keep the volume of the external solution constant. The absorbance of the PBS solution taken out from the released external solution at every interval was recorded with Evolution 201 UV-Vis spectrophotometer to determine the drug concentration in the solution. The cumulative release amount of the drugs was estimated by the concentration of ibuprofen in the solution.

### 2.5. Stability Test and Cytotoxicity Study

The stability test of UiO-66-NH_2_@EPSQ nanocomposites in PBS solution (pH 7.4) was conducted as follows: UiO-66-NH_2_@EPSQ nanocomposites (0.2 g) were well dispersed in 20 mL PBS solution under vigorous stirring and soaked for 1, 3, 5 and 7d at room temperature. The samples were separated by centrifugation, washed twice with water and finally the dry powders were obtained after freeze drying process. FTIR technique was utilized to characterize the stability of the materials after soaking in PBS solution.

The cytotoxicity of nanocomposite A, B, and C was assessed by MTT assay. HeLa cells were provided by Shanghai cell bank (Chinese Academy of Sciences, Shanghai, China). 5 × 10^3^ cells/well of HeLa cells were seeded in 96-well plate and incubated in Dulbecco’s Modified Eagle’s Medium (DMEM) supplemented with 10% fetal bovine serum, 1% Penicillin-Streptomycin for 24 h before treated by the composites. Then, nanocomposite A, B, and C with different concentrations (10, 50, and 100 µg/mL) were added into 100 µL culture medium and co-cultured with HeLa cells for 24 h, 48 h and 72 h respectively. Culture medium without composites was used as a blank control. Subsequently, cells were washed with PBS twice, and added 200 μL culture medium and 0.5 mg/mL MTT solution to each well to be incubated for 4 h. Then the MTT-medium mixture was removed and 150 µL of DMSO was added to each well and vibrated for 10 min to fully dissolve the formazan crystals. The absorbance of the above DMSO solution at 570 nm was measured by a microplate reader (EPOCH2T, Bio-tek, VT, US). The cell viability was calculated as follows: viability (%) = (mean absorbance value of treatment group/mean absorbance value of control) × 100%. All samples were analyzed in triplicate. Cell viability was further analyzed with the Live/Dead cell staining kit (BB4126-1, Best Bio, Shanghai, China). 1 × 10^4^/well of Hela cells were seeded into 24-well plate, and treated by nanocomposite A, B, and C with different concentrations (10, 50, and 100 µg mL^−1^) for 72 h, and stained with 2 μM Calcein-AM and 4 μM PI for 30 min at room temperature. Cells were finally observed with fluorescence microscopy (Leica, DMI8, Weztlar, Germany) and analyzed with Image-Pro Plus software (Media Cybernetics, Bethesda, MD, USA).

## 3. Results and Discussion

### 3.1. Synthesis and Characterization of UiO-66-NH_2_@EPSQ

**Figure 1:**
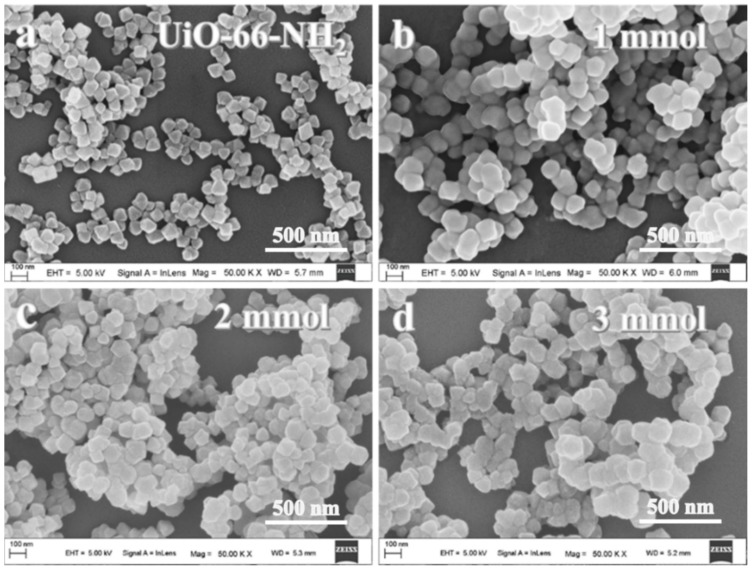
SEM images of (**a**) UiO-66-NH_2_ nanoparticles and (**b**–**d**) UiO-66-NH_2_@EPSQ nanoparticles A (1 mmol), B (2 mmol), and C (3 mmol), respectively. (UiO-66-NH_2_@EPSQ nanoparticles A, B, C stand for 0.2 g UiO-66-NH_2_ coated separately with 1 mmol, 2 mmol, 3 mmol γ-glycidyloxypropyltrimethoxysilane).

**Figure 2:**
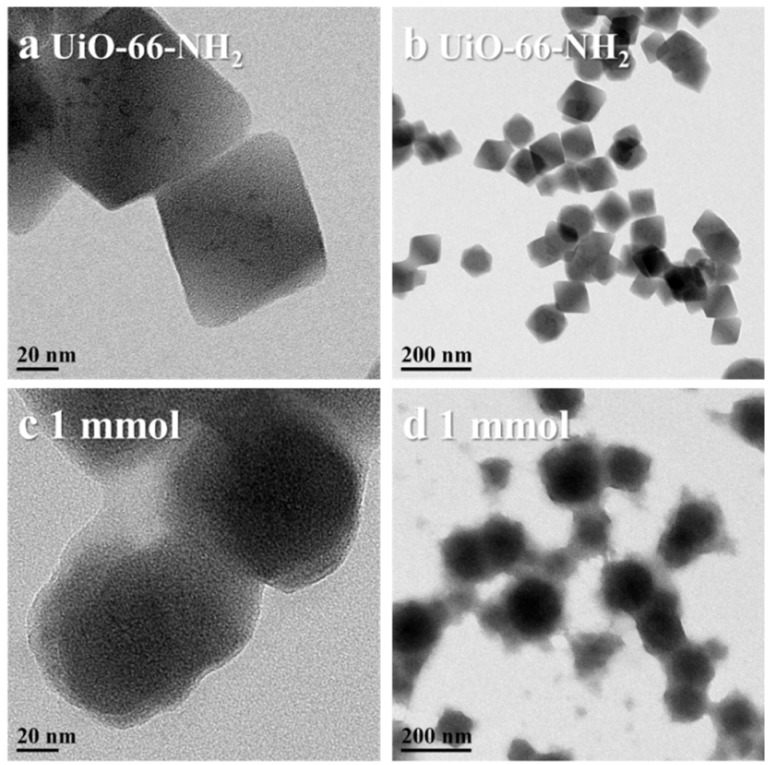
TEM images of (**a**,**b**) UiO-66-NH_2_ nanoparticles and (**c**,**d**) UiO-66-NH_2_@EPSQ nanoparticles.

UiO-66-NH_2_, a kind of MOF built from [Zr_6_O_4_(OH)_4_] octahedron clusters and 2-aminoterephthalic acid ligands, has received considerable attention because of its promising chemical and physical properties and potential applications in drug delivery [50,51,52]. As shown in Figure 1a, UiO-66-NH_2_ nanoparticles with regular octahedron structure and size of approximately 90 nm were prepared via a solvothermal process using ZrCl_4_ reacted with 2-aminoterephthalic acid. UiO-66-NH_2_@EPSQ composites were fabricated by the hydrolysis–condensation of organosilane precursor on the surface of UiO-66-NH_2_ nanoparticles. After coating with the organosilica layer, UiO-66-NH_2_@EPSQ particles with clear spherical shape and diameter of approximately 110–120 nm were obtained with the organosilica shell having a thickness of approximately 10–15 nm (Figure 1b–d). The coating of the organosilica layer on UiO-66-NH_2_ particles was confirmed by the TEM images (Figure 2), with the core shell structure could be clearly figured out.

**Figure 3:**
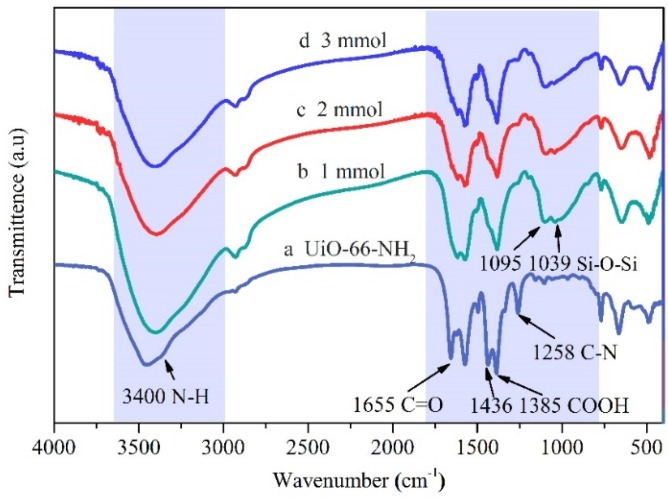
FTIR spectra of UiO-66-NH_2_ and UiO-66-NH_2_@EPSQ nanocomposites.

In the FTIR spectra shown in Figure 3, the characteristic peak at 3400 cm^−1^ is assigned to the –NH_2_ group on UiO-66-NH_2_ particles and the peak at 1258 cm^−1^ represents the stretching vibration of C–N, [53,54], which becomes weaker after wrapping with PSQ. For the spectra of UiO-66-NH_2_@EPSQ (Figure 3b–d), the characteristic absorption peaks of Si–O–Si appear at 1039 and 1095 cm^−1^ [55]. After coating with PSQ, the peak shape of the stretching modes of the carboxylic groups in the NH_2_–BDC ligand at 1436 and 1385 cm^−1^ becomes similar to that of UiO-66, which could be due to the decrease in the –NH_2_ functional group [56]. No obvious peaks were found within 900–980 cm^−1^ for the characteristic absorption peak of epoxy functional, which resulted from the ring-opening reaction occurred between the –NH_2_ group from UiO-66-NH_2_ and the epoxy groups from EPSQ.

**Figure 4:**
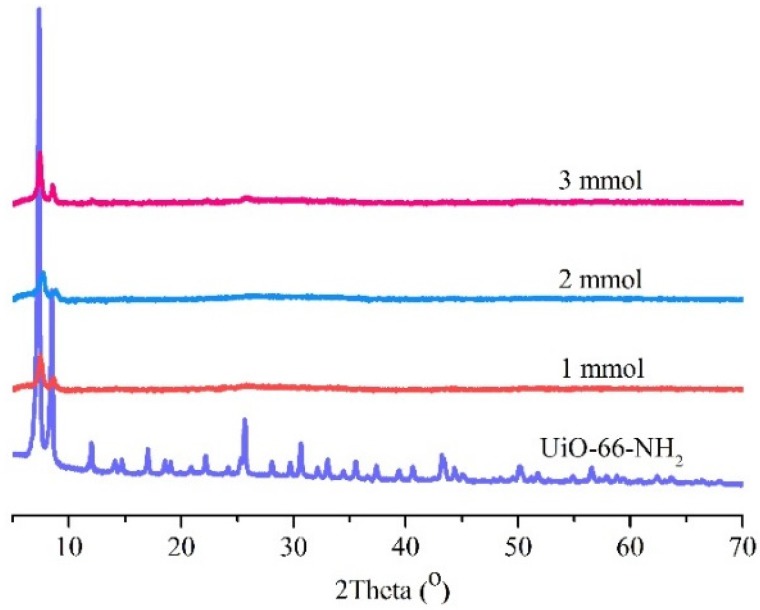
PXRD pattern of UiO-66-NH_2_ and UiO-66-NH_2_@EPSQ nanocomposites.

The XRD pattern demonstrated the high crystallinity of the as-prepared UiO-66-NH_2_ (Figure 4). The strong peaks at −5° and −8° are consistent with the XRD pattern of the UiO-66-NH_2_ framework [57]. The high intensity of the XRD characteristic peaks indicates a highly crystalline structure for UiO-66-NH_2_ synthesized by the solvothermal process. For the XRD pattern of UiO-66-NH_2_@EPSQ, two strong peaks were found within 5°–8°, and all other peaks became weak or disappeared after wrapping with EPSQ. This finding is mainly due to the amorphous organosilica material coated on the outer layer of UiO-66-NH_2_.

### 3.2. Loading and Release Behavior of Ibuprofen with UiO-66-NH_2_@EPSQ as Carriers

Ibuprofen, a typical anti-inflammatory drug, was introduced into the UiO-66-NH_2_@EPSQ nanocomposite to explore its capabilities as a drug carrier. The drug loading characteristics of the particles prepared from different amounts of organosilane precursors are shown in Table 2. The samples showed high drug capacity. Drug loading efficiency (DLE) and drug encapsulation efficiency (DEE) are used to estimate the drug capacity of carriers. DLE is defined as the ratio of the amount of drug in the nanoparticle to the total amount of the nanoparticles [58]. DEE is defined as the ratio of the amount of drug in the nanoparticle to the total amount of drug added for the preparation [59]. For all samples, DLE is basically close to the input ratio. When the input ratio is 50%, the DLE is above 46%; when the input ratio is 100%, the DLE is above 98%; when the input ratio is 200%, the DEL is above 193%. Meanwhile, the DEEs for all samples are basically above 97%. The high drug loading capacity of composite carriers is thought to be partially attributed to the interaction between amino or epoxy groups on the carriers and carboxylic groups on ibuprofen molecules.

(1) $$DLE=\frac{m_{d}-m_{f}}{m_{p}}\times 100\%$$

(2) $$DEE=\frac{m_{d}-m_{f}}{m_{d}}\times 100\%$$

(3) $$E_{r}=\frac{V_{e}\sum_{i=1}^{n-1}C_{i}+V_{0}C_{n}}{m_{d}}$$

In Formulas (1)–(3): DLE: drug loading efficiency, %; DEE: drug encapsulation efficiency, %; *E_r_*: cumulative drug release, %; *m_d_*: total amount of drug added for the preparation, mg; *m_f_*: amount of drug in the nanoparticle, mg; *m_p_*: total amount of nanoparticles, mg; *V_e_*: supplementary PBS solution volume, 3 mL; *C_i_*: drug concentration in the release solution during the ith replacement, mg/mL; *V*_0_: volume of the release solution, mL; *C_n_*: drug concentration in the release solution during the (*i* + 1)th replacement, mg/mL; and *n*: total number of samples.

**Figure 5:**
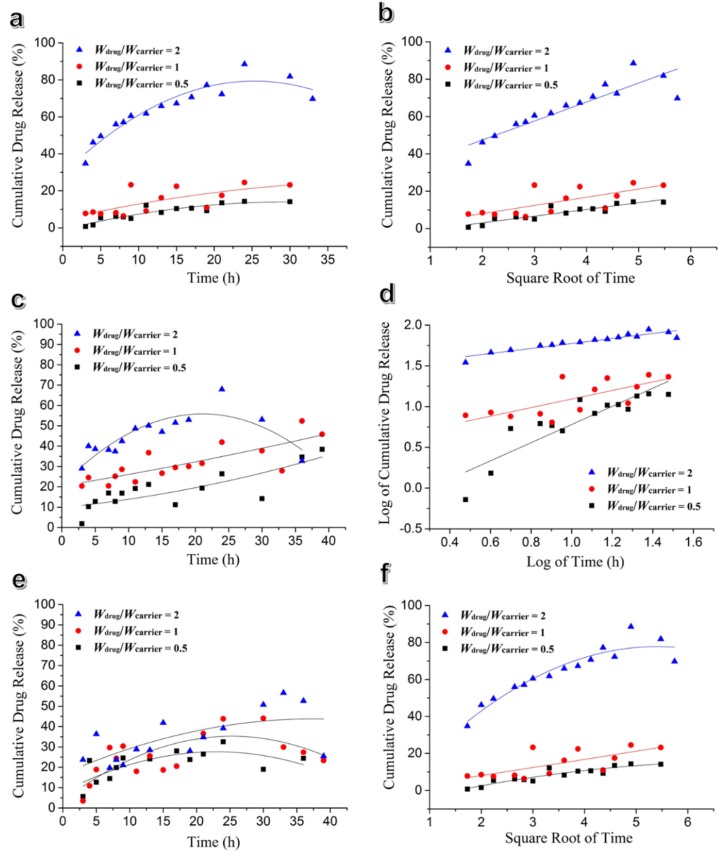
Cumulative release rate–time relationship diagram of the drug loaded with (**a**) nanocomposites A; (**c**) nanocomposites B; and (**e**) nanocomposites C, at different drug doses (blue, red, and black). The kinetic modelling of the drug release from nanocomposites A: (**b**) Higuchi model; (**d**) Korsemeyer-Peppas model; and (**f**) Kopcha model.

Figure 5 shows the release behavior of ibuprofen from UiO-66-NH_2_@EPSQ particles with different nanocomposites (Figure 5a,c,e) at different drug doses (blue, red, and black). The system has a sustained release property. In Figure 5a,c,e, the cumulative release of drugs also increased with the maximum release percentage with increasing drug dosage or approximately 80% for composite A with *W*_drug_/*W*_carrier_ is 2. With increasing silane precursor, that is, the increase in amine, hydroxyl, epoxy, ether bond, and other functional groups, the cumulative release of *W*_drug_/*W*_carrier_ = 2 shows a decreasing trend. In addition, with the increase of the drug loading amount, the release time prolonged to reach the plateau and it is speculated that longer releases and higher release rates could be achieved by increasing the drug loading amount.

Higuchi model [60]: The model assumes release from an insoluble matrix as a time-dependent progression in which Fickian diffusion is presumed:

(4) $$Mt=k\sqrt{t}$$

Korsmeyer-Peppas model [61,62]: The model follows release from a spherical polymeric system in which there may be diffusion or erosion:

(5) $$Mt/M\infty =kt^{n}$$

Kopcha model [63]: The model is used to define the amount of diffusion and erosion and their effects on the release rate:

(6) $$Mt=A\sqrt{t}+Bt$$

in these equations, *Mt* and *M∞* represent the amount of drug dissolved at time t and at infinite time, respectively. The kinetic constants are represented by k.

From Figure 5b,d,f and Table 3, it can be concluded that the ibuprofen drug release kinetics from UiO-66-NH_2_@EPSQ fitted into Higuchi’s mode, indicating that release occurred by diffusion. Correspondingly, a Kopcha’s model fitting displayed high A/B values and small B values, indicating release mechanisms were predominantly based on a diffusion process. In addition, the low cumulative release rate/reloaded to the system are believed due to the interaction force between UiO-66-NH_2_@EPSQ and ibuprofen molecules.

### 3.3. Stability Test

**Figure 6:**
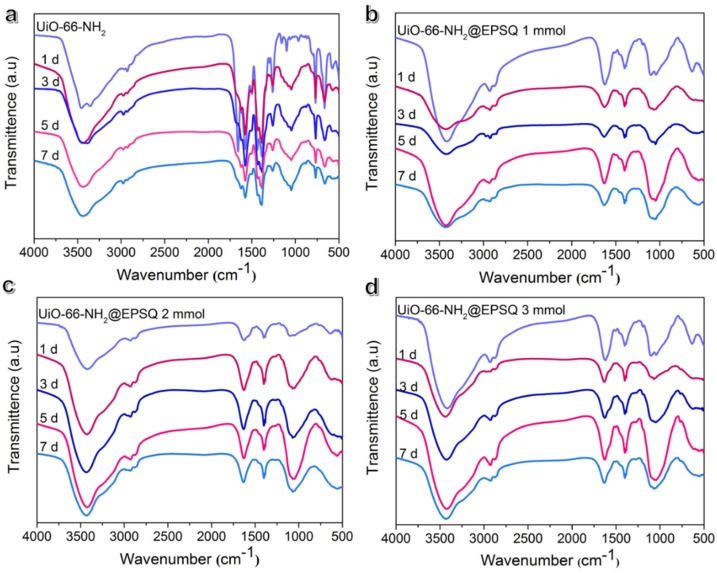
FTIR spectra of (**a**) UiO-66-NH_2_, (**b**) nanocomposites A, (**c**) nanocomposites B, and (**d**) nanocomposites C immersed in PBS solution (pH 7.4) for different times.

Compared with other MOF-based drug carriers, UiO-66-NH_2_ has a high degree of connectivity of the metal clusters in the crystal structure, generating exceptional mechanical stability and chemical stability in a broad range of pH values. In addition, the amino group on the UIO-66-NH_2_ opens up the possibility of post-modification of UiO-66-NH_2_ with multiple properties [64,65,66,67,68]. Meanwhile, polysilsesquioxanes (PSQs), an organosilica with organic/inorganic hybrid structure, have high stability, biocompatibility, and easy functionalization [40,48]. Figure 6 shows the FTIR spectra of UiO-66-NH_2_ and UiO-66-NH_2_@EPSQ nanocomposite A, B, and C after soaking in PBS solution (pH 7.4) at room temperature for a certain period of time. For the sample of UiO-66-NH_2_, after soaking for a week, the absorption peak at 1655 cm^-1^ assigned for C=O obviously decreased sharply (Figure 6a). However, for the FTIR spectra of UiO-66-NH_2_@EPSQ nanocomposites A, B, and C, after soaking for a week, there was no obvious change in each peak. This finding demonstrates that PSQ protected the structure of UiO-66-NH_2_.

### 3.4. Cytotoxicity Test

**Figure 7:**
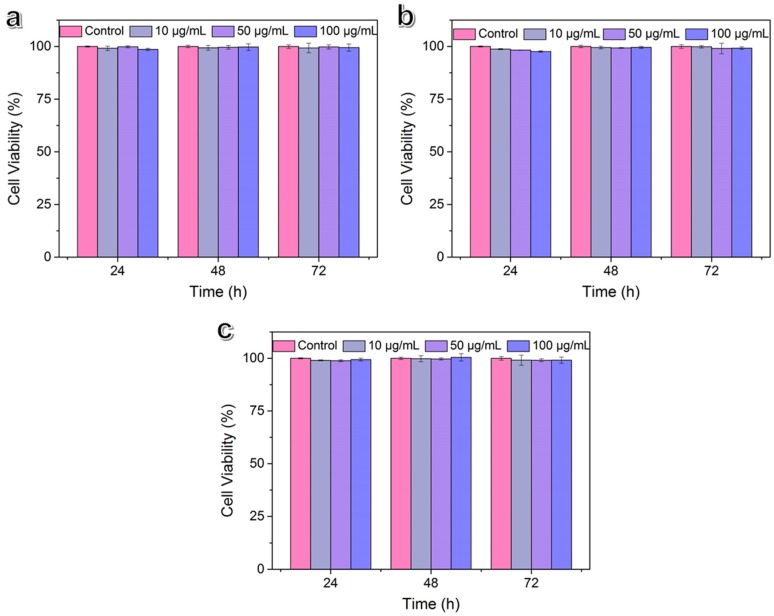
Cytotoxicity test of (**a**) nanocomposite A, (**b**) nanocomposite B, (**c**) nanocomposite C.

**Figure 8:**
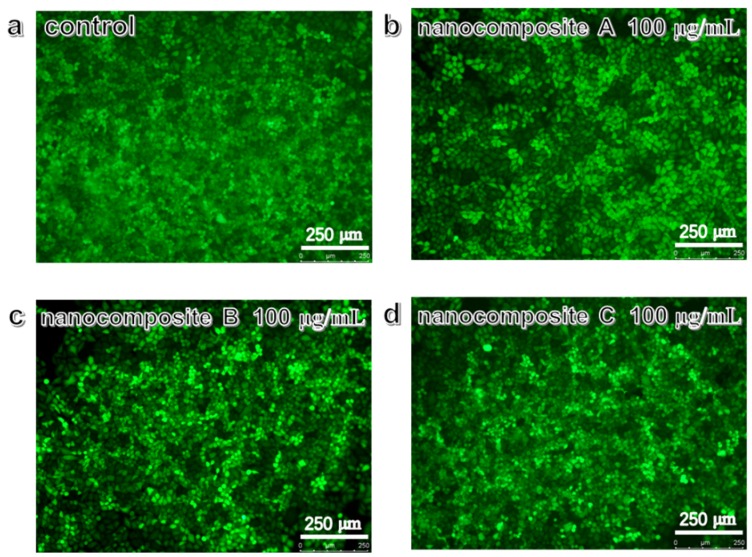
Fluorescent images of Hela cells on (**a**) control plate, (**b**) nanocomposite A, (**c**) nanocomposite B, and (**d**) nanocomposite C for 72 h. Among these, live cells were stained green, while dead cells were stained red.

The biocompatibility of the nanoplatform was evaluated by in vitro cytotoxicity assay. Cytotoxicity was investigated by incubating UiO-66-NH_2_@EPSQ particles with HeLa cells and assessed using MTT assay and live/dead staining assay at different concentrations (10, 50, and 100 µg mL^−1^). As shown in Figure 7, the cell viability remains stable even after incubation for 72 h, regardless of the concentration of the particles. In addition, in Figure 8, there were no dead cells (red fluorescence) even treated by 100 µg mL^−1^ nanocomposites for 72 h. These results clearly demonstrate that the synthesized UiO-66-NH_2_@EPSQ particles have very low cytotoxicity.

## 4. Conclusions

MOF nanocomposites are currently attracting much attention as newly emerging drug carriers. In this study, we reported a drug delivery system based on the nanocomposite of amine-functionalized MOF (UiO-66-NH_2_) embedded with the active organosilica of PSQ layers. The success of the fabrication was confirmed by FTIR, XRD, SEM and TEM characterizations. The obtained organosilica encapsulated MOF (UiO-66-NH_2_@EPSQ) demonstrated high water stability and high drug loading capacity with ibuprofen as a model drug. Ibuprofen from the nanocomposite was also studied. Finally, the preliminary biological studies were performed, which showed that the obtained drug carrier had considerably lower cytotoxicity. We believe that this new nanocomposite delivery system has great potential as a biocompatible system for safe and controlled release of hydrophobic drugs.

## Figures and Tables

**Table 1:** Materials and dosage of drug loading experiment.

Code	*W*_drug_/*W*_carrier_	Ibuprofen	UiO-66-NH_2_@EPSQ
^1^ A1	0.5	0.0020 g	0.0042 g
A2	1.0	0.0041 g	0.0041 g
A3	2.0	0.0081 g	0.0041 g
^2^ B1	0.5	0.0020 g	0.0044 g
B2	1.0	0.0042 g	0.0043 g
B3	2.0	0.0080 g	0.0042 g
^3^ C1	0.5	0.0022 g	0.0043 g
C2	1.0	0.0044 g	0.0043 g
C3	2.0	0.0083 g	0.0042 g

^1^ A1–A3 was loaded with nanocomposites A; ^2^ B1–B3 was loaded with nanocomposites B; ^3^ C1–C3 was loaded with nanocomposites C.

**Table 2:** Drug loading parameters of UiO-66-NH_2_@EPSQ composite with different *W*_drug_/*W*_carrier_ ratios.

i ^1^	*W*_drug_/*W*_carrier_	DLE			DEE		
		Ai	Bi	Ci	Ai	Bi	Ci
		1 mmol	2 mmol	3 mmol	1 mmol	2 mmol	3 mmol
1	50.0%	46.6%	46.6%	50.1%	97.9%	97.9%	97.9%
2	100.0%	98.5%	98.5%	100.1%	98.5%	98.5%	97.9%
3	200.0%	193.1%	193.1%	193.5%	97.7%	97.7%	97.9%

^1^ A1–A3 was loaded with nanocomposites A; B1–B3 was loaded with nanocomposites B; C1–C3 was loaded with nanocomposites C.

**Table 3:** Parameters obtained by fitting the drug release profiles from the UiO-66-NH_2_@EPSQ with kinetic models.

*W*_drug_/*W*_carrier_	Higuchi		Korsemeyer-Peppas		Kopcha			
	R^2^	k	R^2^	n	R^2^	A	B	A/B
0.5	0.8346	0.03595	0.7797	1.1137	0.8418	0.07127	−0.005	−14.254
1	0.4757	0.04387	0.5000	0.5255	0.4233	0.04255	1.8687×10^−4^	227.698
2	0.7890	0.10161	0.8654	0.3072	0.8825	0.32482	−0.03005	−10.809
